# Sex-dependent effects of long-term clozapine or haloperidol medication on red blood cells and liver iron metabolism in Sprague Dawley rats as a model of metabolic syndrome

**DOI:** 10.1186/s40360-021-00544-4

**Published:** 2022-01-15

**Authors:** Marie-Luise Bouvier, Karin Fehsel, Andrea Schmitt, Eva Meisenzahl-Lechner, Wolfgang Gaebel, Martina von Wilmsdorff

**Affiliations:** 1 Department of Psychiatry and Psychotherapy, Medical Faculty, Heinrich-Heine-University, Bergische Landstraße 2, 40629 Düsseldorf, Germany; 2 Department of Psychiatry and Psychotherapy, University Hospital, Ludwig-Maximilians University Munich, Nußbaumstrasse 7, 80336 Munich, Germany; 3 Laboratory of Neuroscience (LIM27), Institute of Psychiatry, University of Sao Paulo, Rua Dr. Ovidio Pires de Campos 785, São Paulo, SP 05453-010 Brazil

**Keywords:** Clozapine, Haloperidol, Blood dyscrasia, Hepatic iron metabolism

## Abstract

**Background:**

Patients with liver diseases often have some form of anemia. Hematological dyscrasias are known side effects of antipsychotic drug medication and the occurrence of agranulocytosis under clozapine is well described. However, the sex-dependent impact of clozapine and haloperidol on erythrocytes and symptoms like anemia, and its association with hepatic iron metabolism has not yet been completely clarified. Therefore, in the present study, we investigated the effect of both antipsychotic drugs on blood parameters and iron metabolism in the liver of male and female Sprague Dawley rats.

**Methods:**

After puberty, rats were treated orally with haloperidol or clozapine for 12 weeks. Blood count parameters, serum ferritin, and liver transferrin bound iron were determined by automated counter. Hemosiderin (Fe^3+^) was detected in liver sections by Perl’s Prussian blue staining. Liver hemoxygenase (HO-1), 5’aminolevulinate synthase (ALAS1), hepcidin, heme-regulated inhibitor (HRI), cytochrome P4501A1 (CYP1A1) and 1A2 (CYP1A2) were determined by Western blotting.

**Results:**

We found anemia with decreased erythrocyte counts, associated with lower hemoglobin and hematocrit, in females with haloperidol treatment. Males with clozapine medication showed reduced hemoglobin and increased red cell distribution width (RDW) without changes in erythrocyte numbers. High levels of hepatic hemosiderin were found in the female clozapine and haloperidol medicated groups. Liver HRI was significantly elevated in male clozapine medicated rats. CYP1A2 was significantly reduced in clozapine medicated females.

**Conclusions:**

The characteristics of anemia under haloperidol and clozapine medication depend on the administered antipsychotic drug and on sex. We suggest that anemia in rats under antipsychotic drug medication is a sign of an underlying liver injury induced by the drugs. Changing hepatic iron metabolism under clozapine and haloperidol may help to reduce these effects of liver diseases.

**Supplementary Information:**

The online version contains supplementary material available at 10.1186/s40360-021-00544-4.

## Background

The atypical antipsychotic drug clozapine is a highly effective drug for use in treatment-resistant schizophrenia. Its risk of agranulocytosis has led to a stringent and mandatory hematologic monitoring over the medication period [1]. Other hematological dyscrasias like eosinophilia, neutropenia, thrombocytopenia, or anemia can also be observed, but the exact pathophysiologic mechanism remains unclear [2]. Anemia is a known but rare side effect of antipsychotic drug medication in patients. The disease is defined as a decrease in the total amount of erythrocytes or hemoglobin content in red blood cells (RBC) associated with a lowered ability to transport oxygen to the cells [3, 4]. The number and volume of erythrocytes, hemoglobin content, mean corpuscular volume (MCV), and red blood corpuscular distribution width (RDW) are the parameters of the blood count to predict anemia and to calculate hematocrit, mean corpuscular hemoglobin (MCH) and mean corpuscular hemoglobin concentration (MCHC). The RDW is a measure of variation in the size of circulating erythrocytes, and can help to differentiate the cause of anemia [5]. A high RDW is often linked to iron, vitamin B_12_ or folate deficiency and to impaired erythropoiesis, thereby reflecting chronic inflammation and increased oxidative stress, both signs of type 2 diabetes [6]. A clinical study [7] found a high incidence of anemia in the first 2 years of clozapine medication. Medical comorbidities like diabetes mellitus enhance the risk to develop anemia under clozapine medication, whereas smoking seems to lower the risk of anemia only in men by upregulating the hemoglobin level in men. In contrast to clozapine, haloperidol rarely causes hematologic side effects like leukopenia [8] or neutropenia [9, 10].

The formation and degradation of erythrocytes are strongly linked to iron metabolism. Hemoglobin is the iron-containing (Fe^2+^) and oxygen-binding red blood pigment in vertebrate erythrocytes with heme as prosthetic group. The liver stores and processes hemoglobin, therefore, people with liver disease often have some form of anemia [11]. However, heme groups are also found in a number of other biologically important hemoproteins as cytochromes, catalases, heme peroxidase, or endothelial nitric oxide synthase. Nearly half of the enzymes of the citric acid cycle and the mitochondrial respiratory chain contain iron or need it as cofactor. The heme groups for these hemoproteins are synthesized in the liver and nonspecific 5-aminolevulinate synthase 1 (ALAS1) is the first and rate-limiting enzyme of this heme biosynthetic pathway in mammalian cells. The heme groups for hemoglobin are synthesized in the bone marrow and erythroid-specific ALAS2 regulates the first step of this heme biosynthesis [12]. Clozapine and haloperidol are metabolized in the liver by the cytochrome P450 (CYP) system. CYP1A2 is the main CYP isoform in clozapine metabolism, and its activity is an important determinant of clozapine dosage [13], whereas CYP1A1 is able to catalyze the metabolism of haloperidol [14].

Iron metabolism is finely regulated to control iron uptake, storage, and recycling [15]. Hepcidin is the main regulator of iron metabolism and is synthesized and released by hepatocytes in response to increased body iron concentration or inflammation [16]. The eukaryotic translation initiation factor 2-alpha kinase 1 (eIF2AK1), also named heme-regulated inhibitor (HRI), acts especially as a heme sensor, and is induced under heme deficiency [17]. In red blood cells, it controls hemoglobin synthesis by regulating the synthesis of heme and globin moieties. However, knockout of HRI increases endoplasmic reticulum (ER) stress, and its pharmacological activation reduces ER-stress-induced hepatic steatosis and glucose intolerance during acute heme deficiency conditions in mouse models [18]. Furthermore, antipsychotic drugs are known to induce injuries in different tissues such as the liver by ER stress, mitochondrial dysfunction or oxidative stress [19]. The oxidative stress-induced heme oxygenase 1 (HO-1), a heme-containing member of the heat shock protein family, has the highest concentrations in spleen and liver and catalyzes the degradation of heme to biliverdin and finally bilirubin, ferrous iron (Fe^2+^), and carbon monoxide.

In our recent studies, we have examined the basic parameters of clozapine and haloperidol medication in serum [20], hepatic [21] and hypothalamic [22] lipid and/or glucose metabolism in a rat model of metabolic syndrome. In this study, we examined the effects of the two antipsychotic drugs on blood parameters and their relation to hepatic iron metabolism.

## Methods

### Ethics statement

All experiments were carried out in accordance with the laws of the local authorities for animal experiments approved by the Landesamt für Natur, Umwelt- und Verbraucherschutz (LANUV) NRW, Postfach 101,052, 45,610 Recklinghausen, Germany; Reference number 9.93.2.10.34.07.227. The study was carried out in compliance with ARRIVE guidelines 2.0.

### Experimental design

Ten-week old 30 male and 30 female healthy Sprague Dawley rats from Taconic (Denmark) housed in 5 groups of 6 same-gender animals (sibling group) from the same litter with free access to water and ground food pellets (maintenance diet (Altromin Spezialfutter GmbH, Germany) with 19% crude protein, 4% crude fat and additionally 15% fat). The animals were maintained on a 12:12 h light/dark cycle at 21 °C and 60% humidity. For acclimatisation the animals were handled every day and weighted 2 times per week from PD 71. From PD 78 the rats were separated from each other and individually housed during the test period. Two animals per sibling group were divided into the control, haloperidol-medicated and clozapine-treated groups, resulting in 10 animals per group. To avoid puberty effects antipsychotic drug treatment started at PD 85 (week 13) to PD 165 (week 25). Male (*n* = 10 per group) or female (*n* = 10 per group) rats were fed orally each day with clozapine (Leponex®, Novartis, Germany with 20 mg/1 kg body weight (BW)/day in ground pellets), corresponding to an effective average dose rate to 18.5 ± 0.26 mg/kg BW for males and 17.7 ± 0.38 mg/kg BW for females [20]. Haloperidol (Haloneurol®, Hexal, Germany) was fed with 1 mg/1 kg BW/day in ground pellets, corresponding to an effective average dose rate of 0.8 ± 0.03 mg/kg BW for males and 0.6 ± 0.08 mg/kg BW for females [20]. Results were compared to those in male (*n* = 10) and female (*n* = 10) control groups fed only with ground pellets. On PD 169 (week 25) twelve hours after food removal, the animals were anaesthetized by Pentobarbital (Narcoren, Merial, Germany), blood samples were collected by aorta puncture and the animals were killed by bleeding out. Serum levels of clozapine and N-desmethylclozapine were quantified by HPLC in the biochemical laboratory of the LVR Klinikum Düsseldorf [20]. Blood count values like leukocytes, erythrocytes, haemoglobin content, hematocrit, mean corpuscular volume (MCV), mean corpuscular haemoglobin (MCH), mean corpuscular hemoglobin concentration (MCHC), red cell distribution width (RDW), thrombocytes, serum ferritin and liver transferrin-bound iron were determined by automated counter in the clinical laboratory of the LVR Klinikum Düsseldorf (Additional file 1).

### Ferric liver iron

Ferric liver iron (Fe^3+^) was analysed by Perl’s Prussian blue staining [23]. Ten μm sections of frozen liver were fixed with 4% buffered neutral formalin at room temperature for 5 min, washed three times with distilled water and air-dried. After descending alcohol series the tissue was prestained in 10% potassium-ferrocyanide for 5 min and stained with equal parts of a mixture of 2% potassium-ferrocyanide solution and 1% hydrochloric acid for 30 min at 37 °C. After washing with distilled water the slides were counterstained with nuclear fast red aluminium sulphate solution (Roth, Germany) for 5 min, rinsed with distilled water, dehydrated in Roti-Histol (Roth, Germany) and mounted in Roti-Histokitt (Roth, Germany). Stained areas were observed under the microscope (Axioscope, Zeiss, Germany). Axiovision software (Zeiss, Germany) recognizes blue coloured hemosiderin deposits and automatically calculate the percentage of the hemosiderin granules in proportion to the uncoloured total area of the image section. This was one on four serial sections and the percental level of hemosiderin was averaged.

### Oxidative stress

Lipid peroxidation in the serum was determined through the production of thiobarbituric acid reactive substances (TBARS) as previously described [24]. 50 μl SDS (8.1%), 375 μl acetic acid (20%, adjusted to pH 3.5 with 1 N NaOH) and 375 μl TBA (0.8% aqueous solution of thiobarbituric acid) were added to 50 μl serum and filled-up to 1 ml with aqua dest. The solution was boiled for 60 min and cooled down to room temperature. 0,5 ml n-butanol + pyridine (15:1) was added, thoroughly mixed, and centrifuged for 10 min at 2000 rpm at room temperature. Spectral absorption was measured at 532 nm and malondialdehyde eqivalents were calculated in μmol/ml by a standard curve.

### Western blot

Protein expression of ALAS1, HO-1, hepcidin, and HRI was identified by Western blot analysis in the hepatic whole cell lysate of each animal, while CYP1A1 and CYP1A2 protein expression were determined in the membrane fraction of each rat. 0.2 g liver was homogenized on ice in 320 μl buffer (0.681 g KH2PO4, 1.1 g Na2HPO4 dissolved in 255 ml aqua dest with protease inhibitor (complete mini, EDTA free, Roche Diagnostics, Germany)) and centrifuged at 12000 g for 10 min at 4 °C.Total protein content of the supernatant (cytosolic fraction) and the pellet (membrane fraction) was measured using the DC Protein Assay from Bio Rad (Germany). 25 μg protein from each animal was separated on NuPAGE™ 4–12% Bis-Tris gels (Thermo Fisher scientific, Germany) and blotted onto Invitrolon membranes (Thermo Fisher scientific). Membranes were then blocked with Roti Block (Carl Roth, Germany) in TBS-T (1:10) at 4 °C for 1 h. The blocked PVDF membranes were incubated overnight at 4 °C either with mouse monoclonal heme oxygenase 1 antibody (1:1000, sc-9390991, Santa Cruz Biotechnology, Germany), goat polyclonal hepcidin antibody (1:1000, sc-240553, Santa Cruz), mouse monoclonal HRI antibody (1:1000, sc-365239, Santa Cruz), rabbit polyclonal ALAS1 (1:1000, ABIN6714659, antibodies-online, Germany), goat polyclonal CYP1A1 antibody (1:2000, ab126887, Abcam, Germany) or mouse monoclonal CYP1A2 antibody (1:1000, sc-53614, Santa Cruz) in TBS-T and then probed at room temperature for 1 h with the HRP-conjugated secondary antibody (1:10000, ab97051, Abcam). Resulting autoradiographs were measured by densitometry using Gene Snap and Gene Tools software (Syngene, Synoptics Ltd., United Kingdom). The membranes were routinely stained with Ponceau-S before immunostaining in order to confirm correct samples loading and the transfer of equivalent amounts of protein [25]. The audioradiographs of each animal were found in the supplementary figure 1.

### Statistical analysis

Our study is an exploratory study, so we chose 10 animals per group (male and female controls, male and female haloperidol-medicated rats, male and female clozapine-medicated animals) to obtain statistically valid conclusions. Statistical analysis was performed using the IBM SPSS version 25 for windows. All data were represented as mean ± SEM and all tests were two-tailed. The data were examined by two-way ANOVA with the between–subject factors MEDICATION and SEX and one-way ANOVA with MEDICATION as factor. In case of significant group effects appropriate posthoc tests were carried out between the groups. Sex-dependent effects were examined by t-tests for independent samples. Due to the small number of animals per group and the explorative character of the study *p*-values < 0.05 were considered as statistically significant and multiple testing was presented without α-adjustment. A bivariate correlation procedure with Spearman-Rho coefficient was carried out to test the relationship between erythrocyte count and hemoglobin, as well as transferrin bound iron (Fe^3+^), the level of Fe^3+^ and hepcidin in liver tissue, ALAS1 and CYP1A1 in the haloperidol groups, and ALAS1 and CYP1A2 in the clozapine groups. CYP1A1 levels were correlated to hemoglobin, aspartate aminotransferase (AST), alanine aminotransferase (ALT), alkaline phosphatase (AP) and the serum level of haloperidol in the haloperidol groups as well as CYP1A2 to hemoglobin, ALT, AP and the serum level of clozapine and desmethyl-clozapine in the clozapine groups with *p < *0.05 as significant. All graphs were performed by Excel 2016 for Windows. Significant correlations are seen as simple scatterplots performed by spss in the supplementary figure 2.

## Results

### **Blood cell count and serum oxidative stress** (Table 1A)

Thrombocytes did not differ between the groups and between male and female rats. Leucocytes varied significantly in SEX [F (1,56) = 14.17 *p* = 0.00043] and MEDICATIONxSEX [F (2,56) = 5.11 *p* = 0.009] and male haloperidol medicated rats differed from female rats (*p* = 0.0003). The number of erythrocytes differed significantly for MEDICATION [F (2,56) = 6.49 *p* = 0.0031] and SEX [F (1,56) = 24.44 *p* = 0.000009]. Female controls had significantly more erythrocytes than female haloperidol medicated rats (*p* = 0.014). Male controls (*p* = 0.002) and haloperidol medicated rats (*p* = 0.00013) had significantly more red blood cells than females. Hemoglobin content differed for males [F (2,28) = 3.49 *p* = 0.045] with a lower hemoglobin content for clozapine medicated rats (*p* = 0.048) and females [F (2,27) = 4.66 *p* = 0.019] with a lower hemoglobin level for haloperidol medicated rats (*p* = 0.037). The hematocrit value differed for MEDICATION [F (2,55) = 3.58 *p* = 0.035] and MEDICATIONxSEX [F (2,55) = 4.63 *p* = 0.014] and female haloperidol medicated rats showed a lower hematocrit than the controls (*p* = 0.034) and the clozapine treated animals (*p* = 0.003). MCV differed for SEX [F (1,56) = 34.93 *p* < 0.000001] and female rats had a higher MCV than the respective males (*p* = 0.00039 for controls, *p* = 0.00022 for clozapine) except the haloperidol treated group. MCH differed for MEDICATION [F (2,56) = 10.24 *p* = 0.00018] and SEX [F (1,56) = 49.85 *p* < 0.000001]; a higher MCH was found in the haloperidol medicated males compared to controls (*p* = 0.001) and clozapine medicated rats (*p* = 0.00022). Female rats had higher MCH values than males (*p* = 0.00004 for controls, *p* = 0.012 for haloperidol, p = 0.001 for clozapine). The MCHC differed for MEDICATION [F (2,56) = 7.17 *p* = 0.002] with higher values of haloperidol medicated females compared to clozapine (*p* = 0.006). RDW differed for MEDICATION [F (2,56) = 5.11 *p* = 0.010], SEX [F (1,56) = 108.46 *p* < 0.000001] and MEDICATIONxSEX [F (2,56) = 7.40 p = 0.002]. A lower RDW value was found in the male haloperidol medicated group (*p* = 0.022), a higher RDW in the male clozapine treated group (*p* = 0.032) and male haloperidol treated rats differed from the clozapine medicated rats (*p* = 0.000027). In all three groups males had a higher RDW than the respective females (*p* < 0.000001 for controls, *p* = 0.045 for haloperidol, p < 0.000001 for clozapine). HbA1c is significantly lowered in the female haloperidol medicated group (*p* = 0.005) [20]. We found no increased malondialdehyde as oxidative stress marker in the serum of antipsychotic drug medicated rats. As expected erythrocyte count was positively correlated to hemoglobin for male (ρ = 0.678 *p* = 0.045) and female (ρ = 0.819 *p* = 0.004) controls, male (ρ = 0.752 *p* = 0.012) and female (ρ = 0.912 *p* = 0.001) clozapine medicated rats and male haloperidol treated animals (ρ = 0.888 p = 0.001), but not for females under haloperidol medication (ρ = 0.159 *p* = 0.683).

**Table 1:**
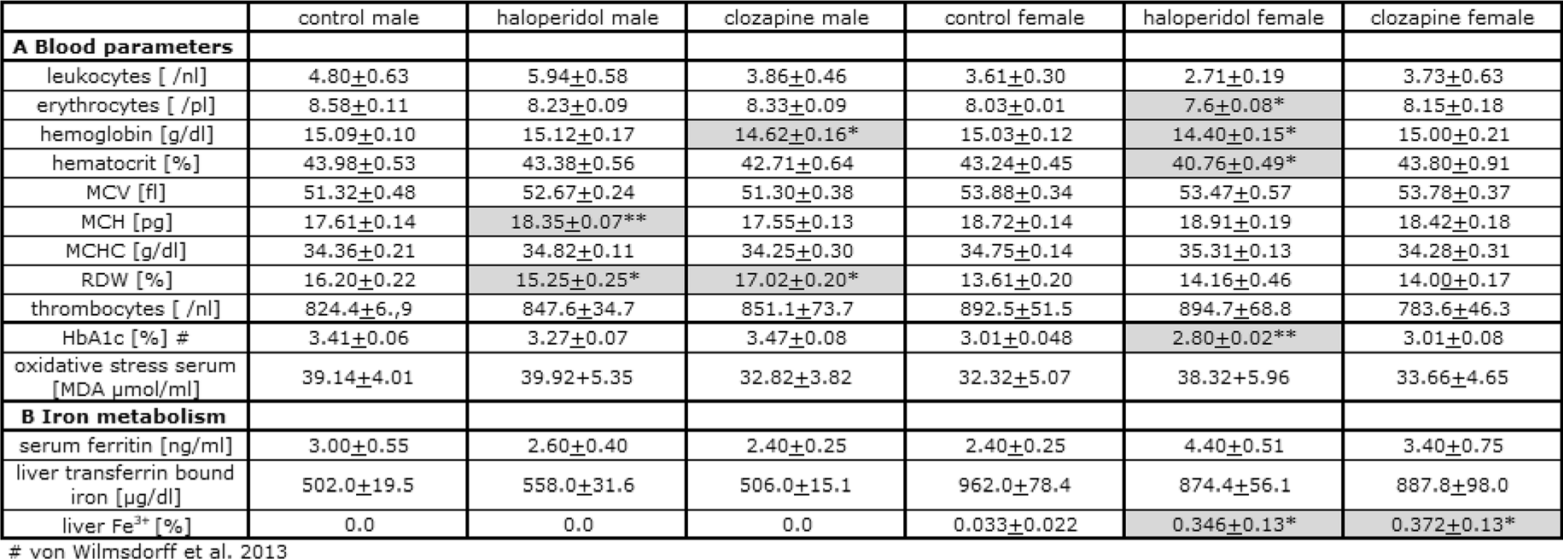
Blood (A) and iron (B) parameters in male and female control, haloperidol and clozapine medicated Sprague Dawley rats after 12 week trial period (*n* = 10). The values marked in grey are significant compared to controls with *p < 0.05 and **p < 0.001 as significant

### **Iron parameters** (Table 1B, Figs. 1 and 2)

**Fig. 1:**
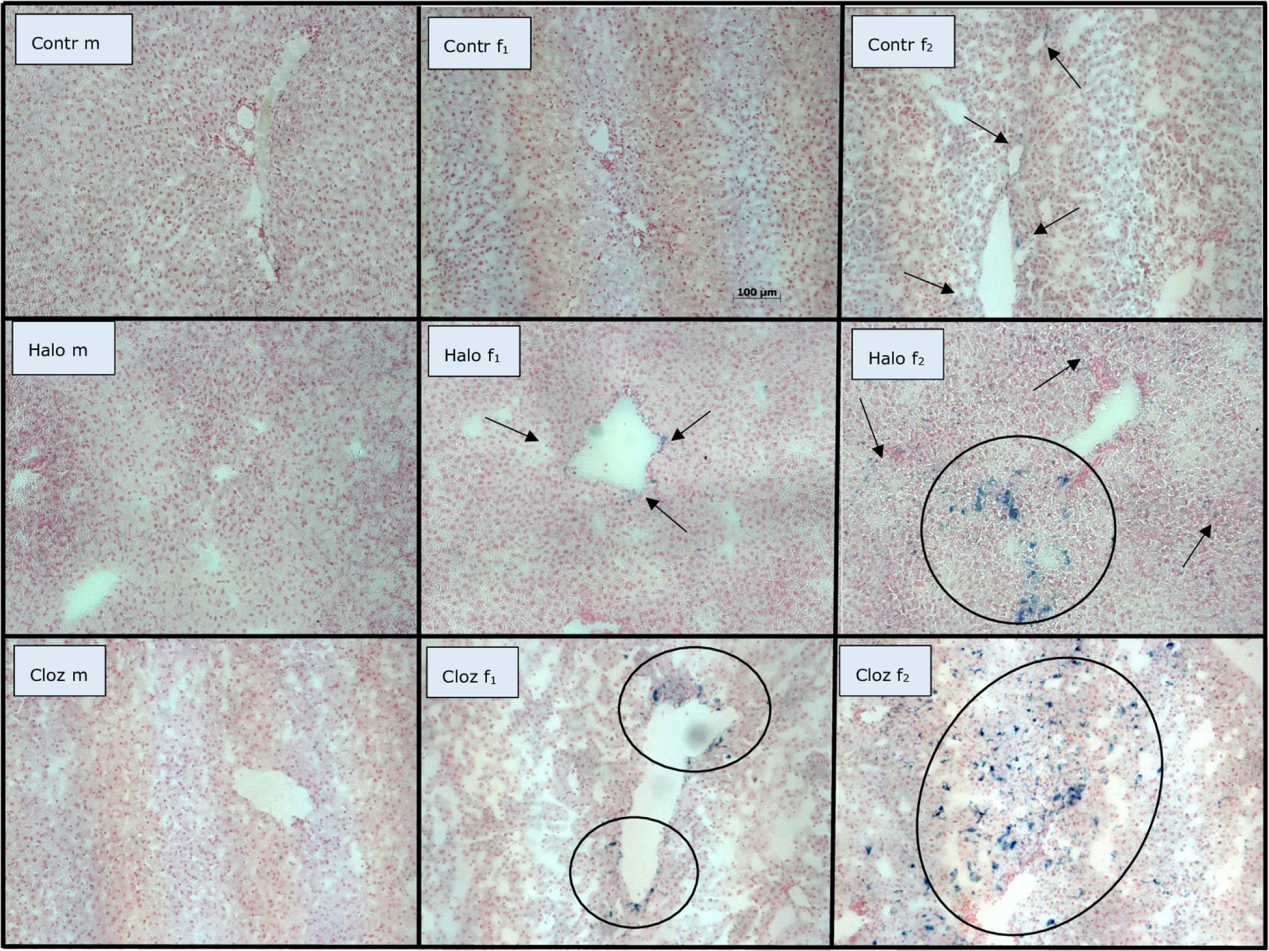
Perl’s Prussian blue staining counterstained with nuclear fast red aluminium sulphate solution on 10 μm frozen liver sections of male and female Sprague Dawley rats after 12 week medication with haloperidol or clozapine, (m = male, f = female, contr = control, Halo = haloperidol medicated, Cloz = clozapine medicated)

Only in female liver tissue, we found the characteristic blue staining of Fe^3+^ by Prussian blue (Table 1B, Fig. 1). Two of the ten control females, six of the ten females under haloperidol medication and six of the eight females medicated with clozapine showed ferric ions (Fe^3+^) in liver tissue with a tenfold higher amount in medicated female rats (*p* = 0.034 for haloperidol, *p* = 0.044 for clozapine). We did not find differences for serum ferritin (Table 1B). Liver transferrin bound iron (Fe^3+^) differed for SEX [F (1,57) = 69.36 *p* < 0.000001], whereas male rats had lower iron levels in liver tissue than the respective females (*p* = 0.00019 for controls, *p* = 0.00010 for haloperidol, *p* = 0.004 for clozapine). No correlation of transferrin bound iron and Fe^3+^ was found in the female groups, but we found a negative correlation between transferrin bound iron and hepcidin in female haloperidol medicated rats (ρ = − 0.929 *p* = 0.001) and between Fe^3+^ and hepcidin in female clozapine medicated rats (ρ = − 0.847 *p* = 0.016).

**Fig. 2:**
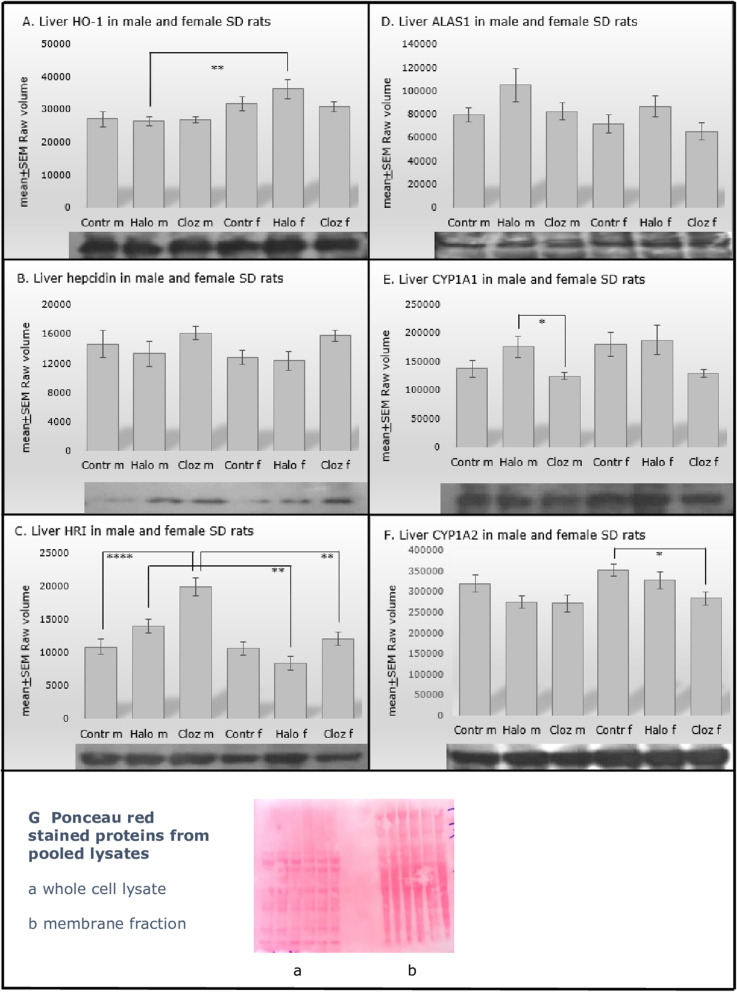
Protein expression of A. heme oxygenase 1 (HO-1), B. hepcidin, C. heme-regulated inhibitor (HRI), D. 5-aminolevulinate synthase 1 (ALAS1), E. cytochrome P450 1A1 (CYP1A1), F. cytochrome P450 1A2 (CYP1A2) in male (m) and female (f) control (Contr), haloperidol (Halo) and clozapine (Cloz) medicated Sprague Dawley rats with *n* = 10 and *p < 0.05, **p < 0.001 and ****p < 0.00001 as significant. The autoradiographs show the protein expression of pooled probes of each treatment group

The level of liver hemoxygenase-1 (Fig. 2A.) did not differ significantly between the controls and the medicated groups, but varied significantly for SEX [F (1,54) = 13.07 *p* = 0.001], in which only haloperidol medicated males and females differed significantly from each other (*p* = 0.014). Hepcidin (Fig. 2B.) did not differ significantly between the groups. HRI (Fig. 2C.) was different for MEDICATION [F (2,54) = 13.03 *p* = 0.00003], SEX [F (1,54) = 25.39 *p* = 0.000007] and MEDICATIONxSEX [F (2,54) = 6.30 *p* = 0.004]. Male clozapine medicated rats differed from male controls (*p* = 0.00005) and from male haloperidol treated rats (*p* = 0.006). Male haloperidol and clozapine medicated rats had higher liver HRI levels than females (*p* = 0.001 for haloperidol and p = 0.001 for clozapine). ALAS1 (Fig. 2D.) did not differ between the groups. CYP1A1 (Fig. 2E.) differed for MEDICATION [F (2,54) = 4.61 *p* = 0.015] and male haloperidol medicated rats showed higher CYP1A1 protein expression than clozapine treated male rats (*p* = 0.05). CYP1A2 (Fig. 2F.) varied for MEDICATION [F (2,54) = 5.18 *p* = 0.009] and SEX [F (1,54) = 4.76 *p* = 0.034], and female rats with clozapine medication differed significantly from the control group (*p* = 0.031).

No correlation was found between CYP1A1 and hemoglobin in the haloperidol-medicated groups and CYP1A2 and hemoglobin in the clozapine treated groups, as well as between ALAS1, CYP1A1 and CYP1A2. Females with haloperidol medication showed a positive correlation of CYP1A1 expression with the liver transaminases AST (ρ = 0.733 *p* = 0.025) and ALT (ρ = 0.783 *p* = 0.013). Male rats, treated with haloperidol, showed a negative correlation between CYP1A1 and the serum level of haloperidol (ρ = − 0.801 *p* = 0.010), whereas males medicated with clozapine showed a positive correlation between CYP1A2 and the serum level of clozapine (ρ = 0.636 *p* = 0.048) as seen in Supplement Fig. 2.

## Discussion

Our study is an explorative examination of clozapine and haloperidol effects on male and female Sprague Dawley rats in the context of the metabolic syndrome. Basic parameters like weight gain, food and water intake, activity, basic serum, hepatic and hypothalamic parameters were showed in previous publications [20–22].

Hematological abnormalities and anemia of diverse etiology are frequently associated with chronic liver diseases [26, 27]. In rats, clozapine induces hepatic oxidative stress leading to liver injury [28] and hepatic steatosis with elevated triglyceride levels [29]. In our study, MCV, measuring the average volume of red blood cells, is unchanged in the medicated groups compared to controls (Table 1). Therefore, we define the observed anemias with decreased overall hemoglobin levels as normocytic. Haloperidol increases the MCH with decreased RDW in male rats (Table 1) associated with high oxidative stress, increased alanine aminotransferase (ALT) and higher neutral fat depots in the liver [21], caused probably by chronic liver disease [30]. In contrast, clozapine decreases hemoglobin with increased RDW in males without changes in the number of erythrocytes, associated with high liver oxidative stress, but without changes of liver transaminases under chronic medication [21]. However, clozapine significantly increases liver mass, neutral fat depots and triglycerides in male hepatic tissue, pointing probably to beginning steatosis. Hepatic fat deposition as sign of liver disease was seen in all medicated groups except for females under clozapine treatment. RDW is a sensitive and specific marker for the assessment of inflammation in patients with non-alcoholic steatohepatitis [11, 31]. Furthermore, high RDW is associated with the metabolic syndrome, a chronic inflammatory disorder and a potential prognostic index for liver disease [32]. Therefore, our results indicate further signs of hepatic non-alcoholic fatty liver disease (NAFLD) in male rats under clozapine medication as seen in our previous studies [20, 21]. In female rats with haloperidol medication, we find decreased erythrocyte counts with lower hemoglobin content, and reduced hematocrit (Table 1), indicating anemia, caused by loss or decreased formation of erythrocytes, and associated with decreased HbA1c levels and elevated hepatic oxidative stress, neutral fat depot and ALT [20, 21]. Interestingly, in the haloperidol group, the erythrocyte count is not correlated with the hemoglobin level as seen in the control and clozapine medicated group (Supplement Fig. 2 A.-D.). It is well known that anemia and red cell turnover affect the Hb1Ac and a low Hb1Ac may reflect decreased liver function as found in US adults without diabetes [33]. In contrast to haloperidol, clozapine does not change the blood count values of females (Table 1). This is in accordance with the study of Wasti et al. [34], who find no characteristic change in red blood cell numbers of clozapine treated rats. However, white blood cells and neutrophil counts are markedly increased with concurrent decrease in lymphocyte count. In summary, we present evidence that the etiopathology of anemia depends on sex and the antipsychotic administered and seems to be an epiphenomenon of an underlying hepatic disease. Male rats under haloperidol medication and female animals under clozapine treatment may have different mechanisms that prevent or at least attenuate the development of anemia. A study [4] in schizophrenic patients under clozapine or haloperidol showed manifestations of anemia pointing to iron deficiency anemia. Unfortunately, this study did not distinguish between sexes. Moreover, a case report showed that hemoglobin levels were severely reduced under clozapine and that clozapine-induced anemia originates from bone marrow suppression [35]. The study by Tanra et al. [36] showed an increase in RDW and mean platelet volume (MPV) under haloperidol, again without differentiation by sex. Iron deficiency anemia, the most common form of anemia, shows a hypochromic, microcytic blood count, whereas the rats in our study are normocytic with decreased hemoglobin levels in male rats like most of the clozapine medicated non-smoker patients [7]. Lee’s study postulates, that anemia under clozapine medication is not based on iron deficiency and therefore anemia under clozapine may represent an epiphenomenon of the underlying psychiatric illness rather than clozapine itself. In contrast, we find anemia with different hematological and hepatic characteristics in male clozapine and female haloperidol medicated animals without psychiatric illness. In consequence, we postulate that hematological dyscrasia under antipsychotic drug medication seems to be a secondary phenomenon of an underlying hepatic injury, caused by the medication itself.

The liver plays a crucial role in iron homeostasis and iron regulation seems to be disturbed in NAFLD [37], which is strongly associated with the metabolic syndrome [38, 39]. Atypical antipsychotics like clozapine can induce the metabolic syndrome and NAFLD [40]. In female Sprague Dawley rats, clozapine and haloperidol lead to non-significantly higher serum ferritin and lower hepatic transferrin bound iron associated with a tenfold higher hepatic ferric ion (Fe^3+^) retention as hemosiderin granules [41], the insoluble form of iron (Table 1, Fig. 1), pointing to iron overload. Interestingly, female controls show also some hemosiderin deposits. The two affected females show metabolic disturbances, such as elevated body weight, increased fat deposition in adipocytes and liver, increased lactate, cholesterol, leptin and iron and decreased insulin, so that the hemosiderin deposits probably result from this. Aged or prematurely damaged erythrocytes are lysed in macrophages, and hemoglobin is degraded to heme and globin. Heme oxygenase (HO-1) in macrophages reduces heme and releases iron, which is quickly exported from the macrophages, bound to plasma transferrin and transported to erythrocyte precursors in the bone marrow, whereas iron, not rapidly released into plasma, is stored in macrophages as ferritin or hemosiderin. Binding of iron as ferritin or hemosiderin decreases the amount of “free iron”, which catalyzes the formation of ROS and oxidizes polyunsaturated phospholipids in membranes of organelles and cells [42]. Our results correspond to the results of Favreau-Lessard et al. [43]. They could show hemosiderin deposition and depleted hepatic glycogen stores as signs of liver injury after doxorubicin medication in mice. In addition to hemosiderin deposition, we have previously found decreased hepatic glycogen stores in clozapine-medicated females [21]. In a rat model of hepatic acute-phase reaction, Malik et al. [44] could show, that increased liver iron may be the consequence of hepatocyte damage. Iron is released into the serum, is then transported back to the liver and stored there as ferritin. In the case of a resulting hyperferritinemia, the iron is then deposited as hemosiderin. Besides hepcidin and HRI, there may be other control mechanisms to control iron metabolism in the two sexes. In the human ferroportin disease, characterized by iron deposits in macrophages, no chronic liver disease was reported, but liver-derived hepcidin levels were elevated, similar to the results in female clozapine treated animals. Furthermore, the retention of iron in hepatic macrophages seems to protect against damage of parenchymal liver cells [45]. Further investigations are needed to explain the hemosiderin deposits and the hepatic glycogen depletion in females. In summary, in the present study, we found a sexual dimorphism to reduce hepatic oxidative stress: females store iron as hepatic hemosiderin granules or ferritin, reflected in higher serum ferritin and liver hemosiderin levels, whereas male clozapine-medicated rats increase the level of hepatic heme regulated inhibitor (HRI) (Fig. 2C.). HRI, the main heme sensor, inhibits excess globin protein or heme synthesis at the translation initiation level in response to oxidative stress or heme deficiency under disease states in hepatocytes, and thus reducing the severity of these diseases [46]. Additionally, HRI controls hepatic P450 cytochromes and decreases ER stress. Its pharmacological activation reduces hepatic steatosis and glucose intolerance in mouse models [17]. In line with these results, the clozapine-treated male rats have normal fasting glucose levels, while the female rats are hyperglycemic [20].

Iron absorption and iron release from cells that recycle or store iron, are regulated by hepcidin [47]. Recent research findings could show that hepcidin levels correlate with liver iron content, but the dysmetabolic iron overload is not necessarily due to altered hepcidin synthesis [48] as seen in haloperidol-medicated females. Females with clozapine medication show non-significant elevation of hepcidin levels (Fig. 2B.), inhibiting probably dietary iron absorption and leading to retention of excess iron as hemosiderin within tissue macrophages [49]. Interestingly, the hepcidin level of female clozapine medicated rats is negatively correlated to the amount of Fe^3+^ in liver tissue, whereas in females under haloperidol medication hepcidin is negatively correlated to the transferrin bound iron in serum (Supplement Fig. 2 E.-F.). Furthermore, hepcidin may enhance hepatic lipid accumulation, as seen in male and female haloperidol and male clozapine treated rats. It seems to play a role in the regulation of hepatic metabolic pathways involved in the pathogenesis of NAFLD [50], but the underlying mechanism is unclear as well as the effect of estrogen, which is known to modulate hepcidin synthesis and serum iron availability [51]. HO-1 and ALAS1 are the rate limiting enzymes of heme degradation and synthesis, respectively. Although HO-1 is stress-induced, its hepatic protein expression (Fig. 2A.) as well as ALAS1 (Fig. 2D.) is not significantly altered in our study by haloperidol or clozapine medication. However, the higher HO-1 expression in haloperidol medicated females could probably help to accumulate iron as a pro-oxidant mechanism [52]. Over 50% of the heme synthesized in the liver is linked to the synthesis of P450 enzymes [53]. However, CYP1A1 (Fig. 2E) is not significantly increased in the haloperidol groups, but is negatively correlated with haloperidol serum level in males (Supplement Fig. 2G.). CYP1A2 (Fig. 2F.) is decreased in both clozapine groups and positively correlated with clozapine serum level in males (Supplement Fig.2H.). Adult male rats seem to be more susceptible to liver injury than females as seen in mice, probably due to decreased expression and/or activity of P450 enzymes [54]. However, the role of estrogen in iron and P450 enzymes metabolism is not yet fully understood [55]. In summary, the synthesis and degradation of hem groups are probably not affected by clozapine and haloperidol medication. However, significant sex-specific differences are found in the expression of HRI and CYP1A2. A possible explication of the metabolic differences between the two sexes could be the fact that clozapine has a lower metabolic conversion rate in male SD rats. Female SD rats, exhibiting no robust weight gain or fat deposition, showed a high level of the metabolite N-desmethyl-clozapine, whereas male SD rats had an equal or higher level of clozapine [20]. In patients, N-desmethylclozapine has higher half-life elimination and possibly contributes significantly to the atypical effects of clozapine treatment by blocking the same receptors [56]. Furthermore, impaired in vitro oxidation of clozapine has been reported in steatotic rat liver due to downregulation of CYP1A [57].

In contrast to haloperidol, which seems to show only rare hematological effects, it is known that clozapine induces dyscrasias of leukocytes [58] and erythrocytes [7] in humans, and chronic liver injury in mice [59] and rats [28, 29]. There is growing evidence that clozapine medication is associated with severe drug-induced hepatic injury in patients [60]. The RDW is a potential prognostic index for liver disease and is increased in rats with hepatic injury associated with the metabolic syndrome [31, 32] and human patients [11]. The liver stores and processes hemoglobin, so people with liver disease often have some form of anemia. Our study has shown that there is evidence from animal models that antipsychotics can lead to pathological liver changes such as steatosis or NAFLD in patients and this is reflected in blood count parameters. An increase in liver transaminases is often seen at the beginning of drug treatment. Closer monitoring of laboratory values such as RDW and hemoglobin may lead to earlier detection of liver damage and thus would reduce morbidity and mortality in patients treated with antipsychotics.

This study provides further information on the sex-dependent metabolic effects of haloperidol and clozapine in blood and iron metabolism, but has several limitations as reported in Bouvier et al. [22]. Briefly, the number of medicated animals is low and further investigations with a higher number of animals per group are needed to ensure our results. Social isolation, essential for the exact medication, is known to be a stress factor, and can influence behaviour and cognitive abilities. Furthermore, we have not considered the effect of iron on glucose metabolism, knowing that iron influences glucose metabolism at multiple levels [61].

## Conclusion

In summary, we found anemia in the blood count of male rats under clozapine medication and female rats under haloperidol medication with varying causes depending on the different drugs. We suggest that the development of anemia is linked to pathological changes in the liver. The oxidative stress under clozapine in the liver tissue associated with high triglyceride levels in males leads to the activation of HRI in males and to the deposition of Fe^3+^ as hemosiderin and degradation of glycogen to glucose in females. Pathological liver changes under antipsychotic medication are shown in animal models, but also in the clinical setting. We provide further evidence that clozapine induces liver steatosis or NAFLD in male rats as recently found in patients under clozapine medication [60]. Further investigations in the clinical field must clarify whether the experimental results in the rat model are valid in human patients. Greater attention to anemia pointing to liver injuries would be helpful.

## Supplementary Information

**Additional file 1.** Supplementary figure 1.

**Additional file 2.** Supplementary figure 2.

## Data Availability

The datasets used and/or analysed during the current study are available from the corresponding author on reasonable request.

## Abbreviations

CBC: Complete blood cell count
RBC: Red blood cells
RDW: Red blood corpuscular distribution width
MCV: Mean corpuscular volume
MCH: Mean corpuscular hemoglobin
MCHC: Mean corpuscular hemoglobin concentration
Hb1Ac: Hemoglobin1Ac
AST: Aspartate aminotransferase
ALT: Alanine aminotransferase
AP: Alkaline phosphatase
NAFLD: Non-alcoholic fatty liver disease
ROS: Reactive oxygen species
TBA: Thiobarbituric acid
MDA: Malondialdehyde
TBAR: Thiobarbituric acid reactive substances
ER: Endoplasmic reticulum
HO: Hemoxygenase
ALAS: 5-aminolevulinate synthase
HRI: Heme regulated inhibitor
eIF2AK: Translational initiation factor 2-alpha kinase = HRI
CYP: Cytochrome P450
PD: Postnatal day

## References

[1] Wicinski M, Weclewicz M (2018). Clozapine-induced agranulocytosis/ granulocytopenia: mechanisms and monitoring. Curr Opin Hematol.

[2] Peinado AG, Ruiz PC, Fraile SC, Cano MG, Fajardo GEB (2016). Clozapine induced blood dyscrasias and a therapeutical approach. Eur Psychiatr.

[3] Wasti A, Ghani R, Manji MA, Siddiqui NA (2004). Haloperidol induced variations in hematological indices. Pak J Med Sci.

[4] Wasti A, Zahid S, Ahmed N (2013). Antipsychotic drugs induced iron deficiency anemia in schizophrenic patients. Int J Advanced Res.

[5] May JE, Marques MB, Reddy VVB, Gangaraju R (2019). Three neglected numbers in the CBC: the RDW, MPV, and NRBC count. Cleveland Clin J Med.

[6] Dada OA, Uche E, Akinbami A, Odesanya M, John-Olabode S, Adediran A, Oshinaike O, Ogbera AO, Okunoye O, Arogundade O, Aile K, Ekwere T (2014). The relationship between red blood cell distribution width and blood pressure in patients with type 2 diabetes mellitus in Lagos. Nigeria J Blood Med.

[7] Lee J, Bies R, Bhaloo A, Powell V, Remington G (2015). Clozapine and anemia. A 2-year follow-up study. J Clin Psychiatr.

[8] Huang W, Lin C, Liao S (2011). Decreased white blood cell count related to haloperidol add-on treatment to olanzapine. Taiwan J Psychiatr.

[9] Flanagan RJ, Dunk L (2008). Haematological toxicity of drugs used in psychiatry. Hum Psychopharmacol Clin Exp.

[10] Sahan E (2016). Haloperidol.related neutropenia. Indian J Psychiatr.

[11] Hu Z, Sun Y, Wang Q, Han Z, Huang Y, Liu X, et al. Red blood cell distribution width is a potential prognostic index for liver disease. Clin Chem Lab Med. 2013;51(7). 10.1515/cclm-2012-0704. 10.1515/cclm-2012-0704 23314558

[12] Kubota Y, Nomura K, Katoh Y, Yamashita R, Kaneko K, Furuyama K (2016). Novel mechanisms for heme-dependent degradation of ALAS 1 protein as a component of negative feedback regulation of heme biosynthesis. J Biol Chem.

[13] Dean L. Clozapine therapy and CYP2D6, CYP1A2, and CYP3A4 genotypes. In: Pratt V, McLeod H, Dean L, et al., editors. Medical Genetics Summaries (Internet). Bethesda (MD): National center for Biotechnology; 2016.

[14] Fang J, McKay G, Song J, Remillrd A, Li X, Midha K (2001). In vitro characterization of the metabolism of haloperidol using recombinant cytochrome P450 enzymes and human liver microsomes. Drug Metab Dispos.

[15] Rivella S, Crielaard BJ. Disorders of iron metabolism: iron deficiency and iron overload and anemia of chronic diseases. In: McManus L, Mitchell RN, editors. Pathobiology of human diseases: Elsevier, Amsterdam, Netherlands; 2014. p. 1471–87.

[16] Silvestri L, Nai A, Dulja A, Pagani A (2019). Hepcidin and the BMP-SMAD pathway: an unexpected liaison. Vitam Horm.

[17] Burwick N, Aktas BH (2017). The eIF2-alpha kinase HRI: a potential target beyond the red blood cell. Expert Opin Ther Targets.

[18] Zarei M, Barroso E, Leiva R, Barniol-Xicota M, Pujol E, Escolano E (2016). Heme-regulated eIF2alpha kinase modulates hepatic FGF21 and it activated by PPARbeta/delta deficiency. Diabetes.

[19] Foufelle F, Fromenty B (2016). Role of endoplasmatic reticulum stress in drug-induced toxicity. Pharma Res Per.

[20] von Wilmsdorff M, Bouvier ML, Henning U, Schmitt A, Schneider-Axmann T, Gaebel W (2013). The sex-dependent impact of chronic clozapine and haloperidol treatment on characteristics of the metabolic syndrome in a rat model. Pharmacopsychiatry.

[21] von Wilmsdorff M, Bouvier ML, Henning U, Schmitt A, Schneider-Axmann T, Gaebel W (2014). Sex-dependent metabolic alterations of rat liver after 12-week exposition to haloperidol or clozapine. Horm Metab Res.

[22] Bouvier ML, Fehsel K, Schmitt A, Meisenzahl-Lechner E (2020). Gaebel W, von Wilmsdorff M (2020) sex-dependent alterations of dopamine receptor and glucose transporter density in rat hypothalamus under long-term clozapine and haloperidol medication. Brain Behav.

[23] Romeis B. Mikroskopische Technik (17. neubearbeitete und erweiterte Auflage, herausgegeben von P. BÖCK). Urban und Schwarzenberg 1989, München – Wien – Baltimore. ISBN: 3‐541‐11227‐1.

[24] Ohkawa H, Ohishi N, Yagi K (1979). Assay for lipid peroxides in animal tissues by thiobarbituric acid reaction. Anal Biochem.

[25] Romero-Calvo I, Ocon B, Martinez-Moya P, Suarez MD, Zarzuelo A, Martinet-Augustin O, de Medina FS (2010). Reversible Ponceau staining as a loading control alternative to actin in Western blots. Anal Biochem.

[26] Gonzalez-Casas R, Jones EA, Moreno-Otero R (2009). Spectrum of anemia associated with chronic liver disease. World J Gastroenterol.

[27] Gkamprela E, Deutsch M, Pectasides D (2017). Iron deficiency anemia in chronic liver disease: etiopathogenesis, diagnosis and treatment. Ann Gastroenterol.

[28] Zlatkovic J, Todorovic N, Tomanovic N, Boskovic M, Djordjevic S, Lazarevic-Pasti T (2014). Chronic administration of fluoxetine or clozapine induces oxidative stress in rat liver: a histopathological study. Europ J Pharmaceut Sci.

[29] Li Y, Su R, Xu S, Huang Q, Xu H (2017). Artesunate prevents rats from the clozapine-induced hepatic steatosis and elevation in plasma triglycerides. Neuropsychiatr Dis Treat.

[30] Das SK, Mukherjee S, Vasudevan DM, Balakrishnan V (2011). Comparison of haematological parameters in patients with non-alcoholic fatty liver disease and alcoholic liver disease. Singap Med J.

[31] Dogan S, Celikbilek M, Zararsiz G, Demiz K, Sivgin S, Guven K (2016). Red blood cell distribution width as a non-invasive marker for the assessment of inflammation in non-alcoholic steatohepatitis. Hepatogasteroenterology.

[32] Laufer Perl M, Havakuk O, Finkelstein A, Halkin A, Revivo M, Elbaz M, Herz I, Keren G, Banai S, Arbel Y (2015). High red blood cell distribution width is associated with the metabolic syndrome. Clin Hemorheol Microcirc.

[33] Carson AP, Fox CS, McGuire DK, Levitan EB, Laclaustra M, Mann DM, Muntner P (2010). Low hemoglobin A1c and risk of all-cause mortality among US adults without diabetes. Circ Cardiovasc Qual Outcomes.

[34] Wasti A, Ghani R, Manji MA, Ahmed N (2006). Clozapine induced neutrophil cytotoxicity in rats. J Pak Med Assoc.

[35] Eleftheriou G, Butera R, Barcella L, Falanga A (2020). Clozapine-induced anemia: a case-report. Int J Clin Pharmacol Ther.

[36] Tanra AJ, Hawaidah M, Mahri Y, Syamsuddin S, Usman AN, Lisal ST (2019). The side effect of haloperidol in schizophrenic patients: analysis of red blood cell distribution width (RDW) and mean platelet volume (MPV) values. Global J Health Sci.

[37] Milic S, Mikolasevic I, Orlic L, Devcic E, Starcevic-Cizmerevic N, Stimac D (2016). The role of iron overload in chronic liver disease. Med Sci Monit.

[38] Hamaguchi M, Kojima T, Takeda N, Nakagawa T, Taniguchi H, Fujii K, Omatsu T, Nakajima T, Sarui H, Shimazaki M, Kato T, Okuda J, Ida K (2005). The metabolic syndrome as a predictor of nonalcoholic fatty liver disease. Ann Intern Med.

[39] Watanabe S, Yaginuma R, Ikejima K, Miyazaki A (2008). Liver diseases and metabolic syndrome. J Gastroenterol.

[40] Xu H, Zhuang X (2019). Atypical antipsychotics-induced metabolic syndrome and non-alcoholic fatty liver disease: a critical review. Neuropsychiatr Dis Treat.

[41] Guindi M, Saxena R (2011). Liver disease in iron overload. practical hepatic pathology: a diagnostic approach.

[42] Ramm GA, Ruddell RG (2005). Hepatotoxicity of iron overload: mechanisms of iron-induced hepatic fibrogenesis. Semin Liver Dis.

[43] Favreau-Lessard A, Blaszyk H, Jones MA, Sawyer DB, Pinz IM (2019). Systemic and cardiac susceptibility of immune compromised mice to doxorubicin. Cardio-Oncology.

[44] Malik IA, Wilting J, Ramadori G, Naz N (2017). Reabsorption of iron into acutely damaged rat liver: a role for ferritins. W J Gastroenterol.

[45] Zoller H, McFarlane I, Theurl I, Stadlmann S, Nemeth E, Oxley D, Ganz T, Halsall DJ, Cox TM, Vogel W (2005). Primary iron overload with inappropriate hepcidin expression in V162del ferroportin disease. Hepatology.

[46] Chen JJ (2007). Regulation of protein synthesis by the heme-regulated eIF2α kinase: relevance to anemias. Blood.

[47] Sangkhae V, Nemeth E (2017). Regulation of the iron homeostatic hormone hepcidin. Amer Soc Nutr.

[48] Marmur J, Beshara S, Eggertsen G, Onelöv L, Albiin N, Danielsson O, Hultcrantz R, Stål P (2018). Hepcidin levels correlate to liver iron content, but not steatohepatitis, in non-alcoholic fatty liver disease. BMC Gastroenterol.

[49] Sebastiani G, Wilkinson N, Pantopoulos K. Pharmacological targeting of the hepcidin/ferroportin axis. Front Pharmacol. 2016; 7: article 160. 10.3389/fphar. 2016.00160. eCollection 2016. 10.3389/fphar.2016.00160 PMC4914558 27445804

[50] Lu S, Bennett RG, Kharbanda KK, Harrison-Findik DD (2016). Lack of hepcidin expression attenuates steatosis and causes fibrosis in the liver. World J Hepatol.

[51] Bajbouj K, Shafarin J, Allam H, Madkour M, Awadallah S, El-Serafy A (2018). Elevated levels of estrogen suppress hepcidin synthesis and enhance serum iron availability in premenopausal women. Exp Clin Endocrinol Diabetes.

[52] Khan ZA, Barbin YP, Cukiernik M, Adams PC, Chakrabarti S (2004). Heme-oxygenase-mediated iron accumulation in the liver. Can J Physiol Pharmacol.

[53] Correia MA, Sinclair PR, De Matteis F (2011). Cytochrome P450 regulation: the interplay between its heme and apoprotein moieties in synthesis, assembly, repair, and disposal. Drug Metab Rev.

[54] Bao Y, Wang P, Shao X, Zhu J, Xiao J, Shi J, Zhang L, Zhu HJ, Ma X, Manautou JE, Zhong XB (2020). Acetaminophen-induced liver injury alters expression and activities of cytochrome P450 enzymes in an age-dependent manner in mouse liver. Drug Metab Dispos.

[55] Hamad M, Bajbouj K, Taneera J (2020). The case for an estrogen-iron axis in health and disease. Exp Clin Endocrinol Diabetes.

[56] Natesan S, Reckless GE, Barlow KB, Nobrega JN, Kapur S (2007). Evaluation of N-desmethylclozapine as a potential antipsychotic-preclinical studies. Neuropsychopharmacology.

[57] Li Z, Lee SH, Jeong HJ, Kang HE (2021). Pharmacokinetic changes of clozapine and norclozapine in a rat model of non-alcoholic fatty liver disease induced by orotic acid. Xenobiotica.

[58] Capllonch A, de Pablo S, de la Torre A, Morales I (2016). Increase in white cell and neutrophil counts during the first eighteen weeks of treatment with clozapine in patients admitted to a long-term psychiatric care inpatient unit. Rev Psiquiatr Salud Ment.

[59] Bai HY, Feng S (2017). Protections effects of schizandrin B against liver injury induced in mice. Yao Xue Xue Bao.

[60] Druschky K, Toto S, Bleich S, Baumgärtner J, Engel RR, Grohmann R, Maier HB, Neyazi A, Rudolph YJ, Rüther E, Schwörer H, Seifert J, Stübner S, Degner D (2020). Severe drug-induced liver injury in patients under treatment with antipsychotic drugs: data from the AMSP study. World J Biol Psychiatr.

[61] Fernandez-Real JM, McClain D, Manco M (2015). Mechanisms linking glucose homeostasis and iron metabolism toward the onset and progression of type 2 diabetes. Diabetes Care.

