# Supplementary material for: Sex-dependent effects of long-term clozapine or haloperidol medication on red blood cells and liver iron metabolism in Sprague Dawley rats as a model of metabolic syndrome
Source: BMC Pharmacol Toxicol. 2022 Jan 15;23:8. doi: 10.1186/s40360-021-00544-4 (PMC8760835; doi:10.1186/s40360-021-00544-4)

Supplementary figure 1: Autoradiographs of every animal of CYP1A1 and CYP1A2 in the membrane fraction, HO-1, HRI, Hepcidin and ALAS-1 in the whole cell lysate of rat liver tissue. The membrane were stripped with Abcam mild stripping buffer and reprbed with the respective primary antibody.


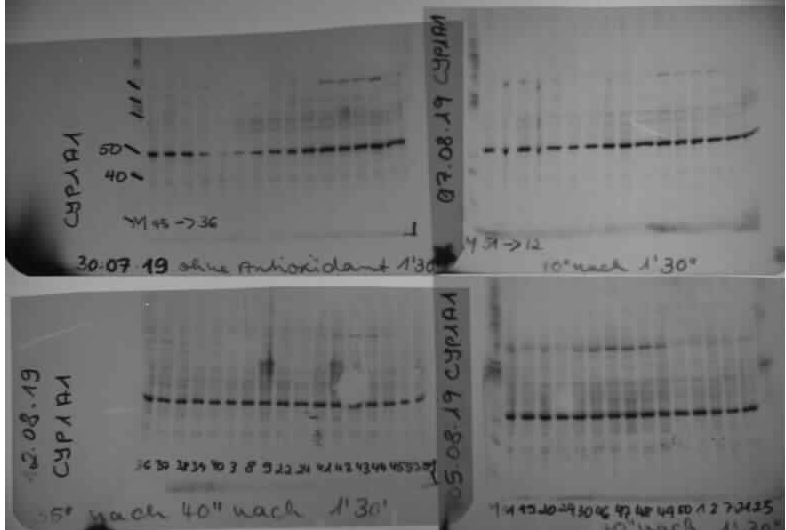

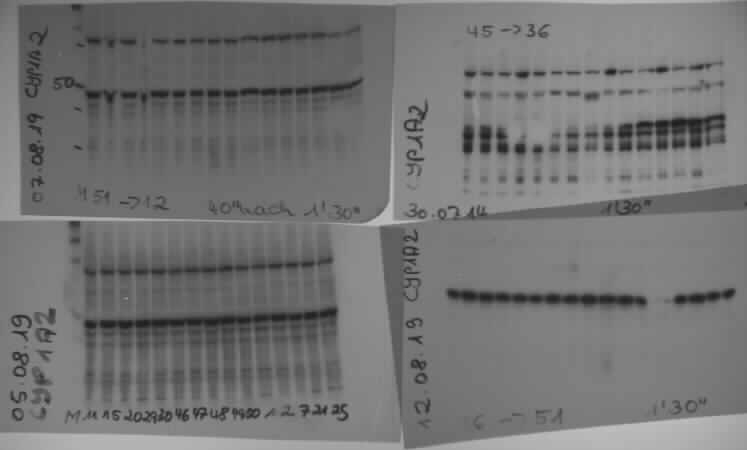


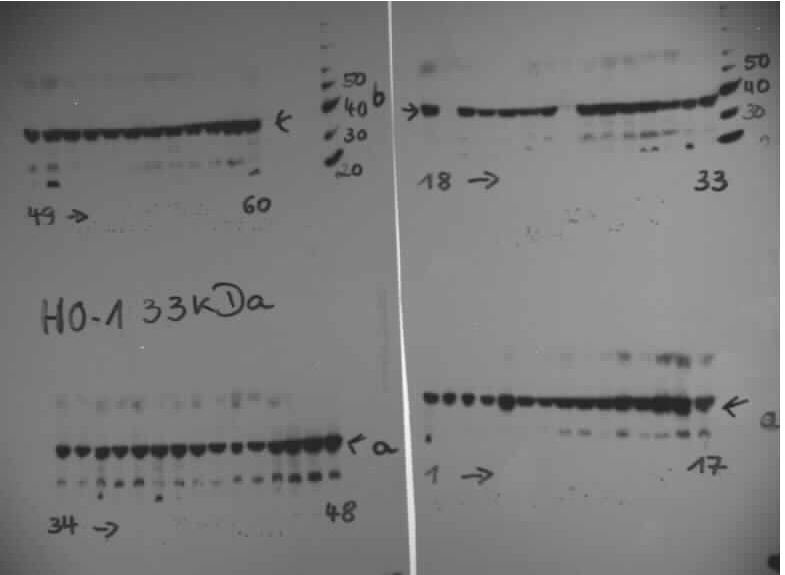

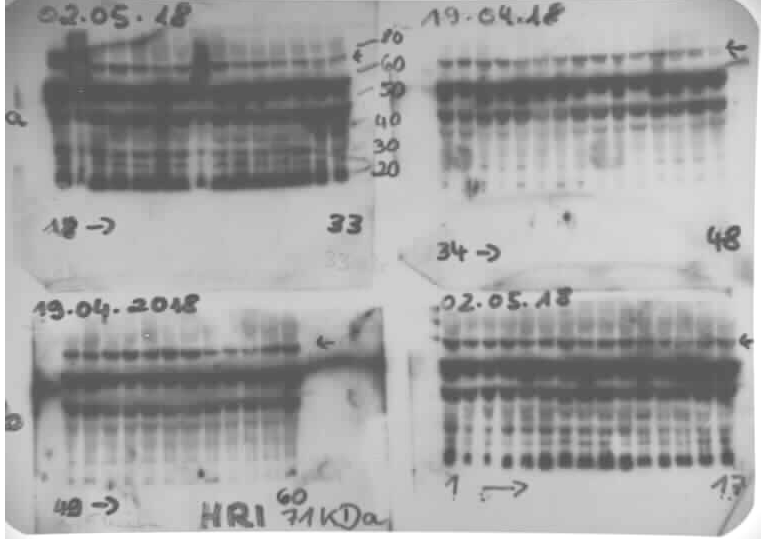

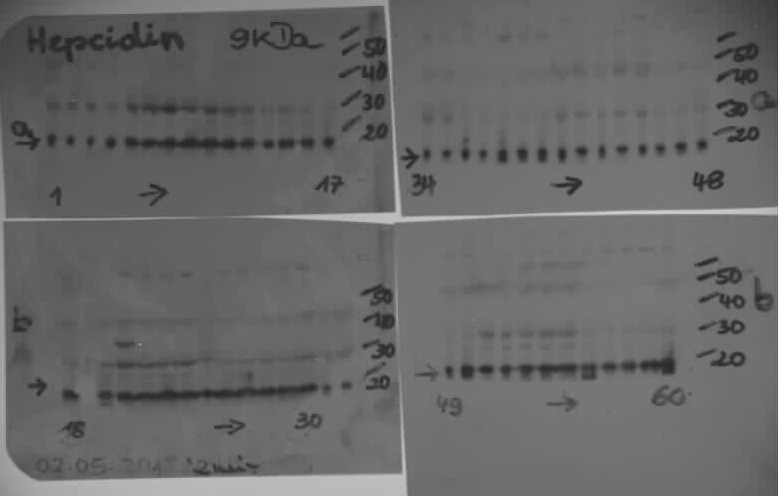


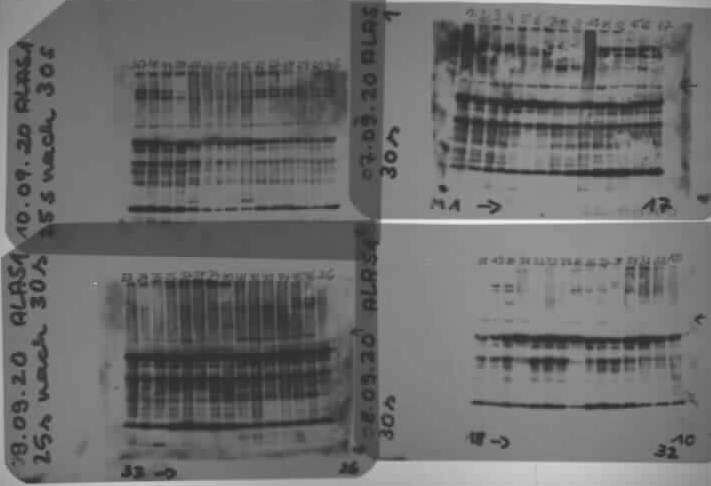

Supplement: Supplementary file 1 — Additional file 1. Supplementary figure 1. [file 40360_2021_544_MOESM1_ESM.docx]
