# Supplementary material for: Sex-dependent effects of long-term clozapine or haloperidol medication on red blood cells and liver iron metabolism in Sprague Dawley rats as a model of metabolic syndrome
Source: BMC Pharmacol Toxicol. 2022 Jan 15;23:8. doi: 10.1186/s40360-021-00544-4 (PMC8760835; doi:10.1186/s40360-021-00544-4)

**Supplementary figure 2: Simple scatterplots of the significant correlations (see result part)**

A.-D. Hemoglobin level (abscissa) versus erythrocyte count (ordinate) of male and female controls and clozapine medicated SD rats

1. Male controls


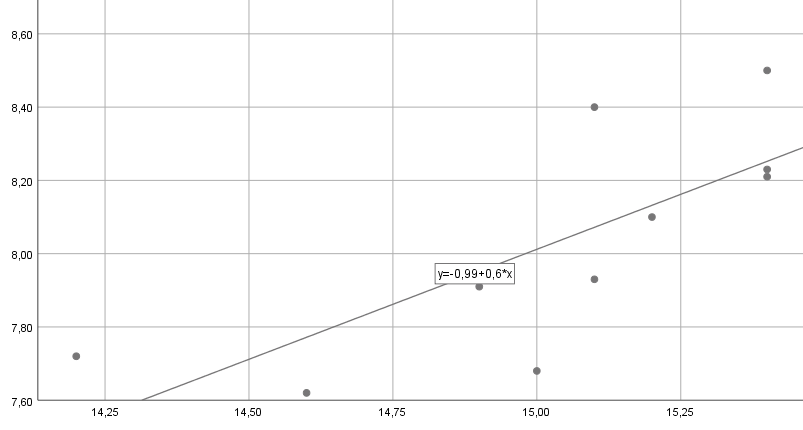


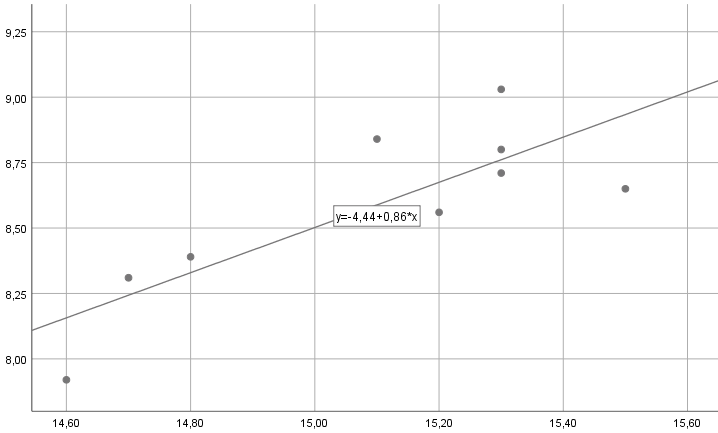


B. Female controls

1. Male controls

D.Female clozapine medicated

C. Male clozapine medicated


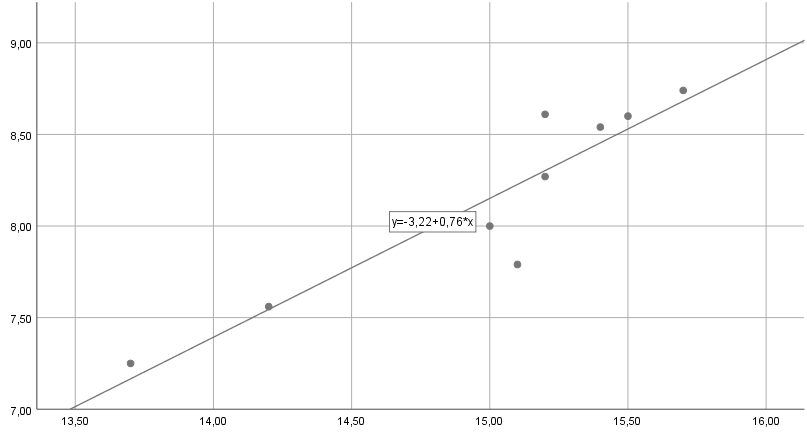


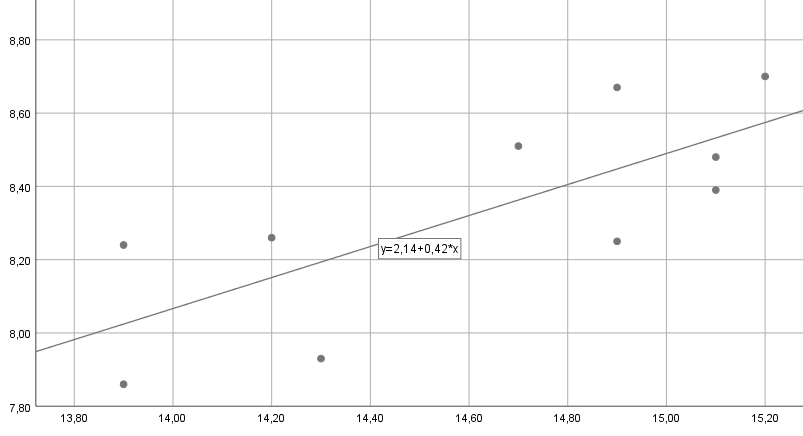


E. Transferrin (abscissa) versus hepcidin (ordinate) of female haloperidol medicated SD rats

F. Percental hemosiderin versus hepcidin hemosiderin of female clozapine medicated SD rats

F. Female clozapine medicated


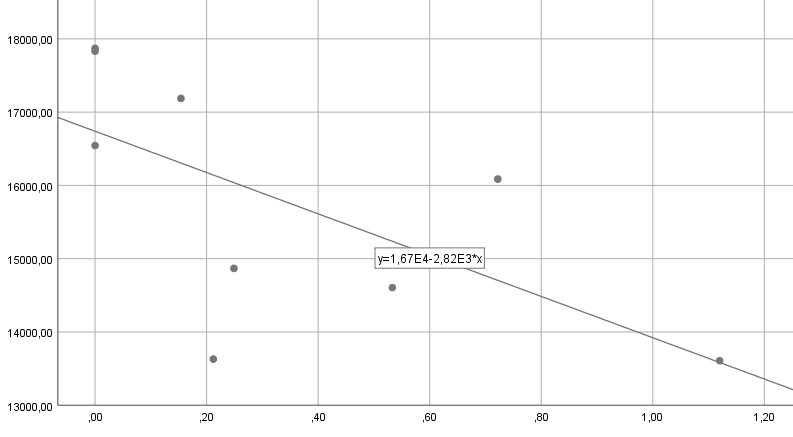


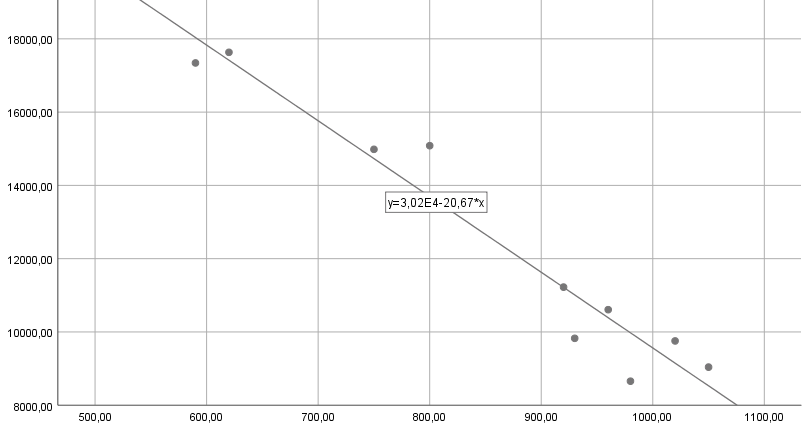


E. Female haloperidol medicated

G. Serum haloperidol level (abscissa) versus CYP1A1 raw volume (ordinate) male haloperidol medicated SD rats

H. Serum clozapine level (abscissa) versus CYP1A2 raw volume (ordinate) of male clozapine medicated SD rats


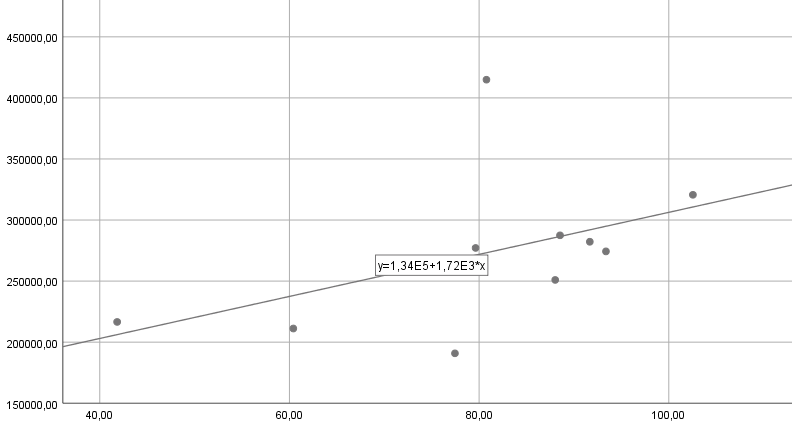


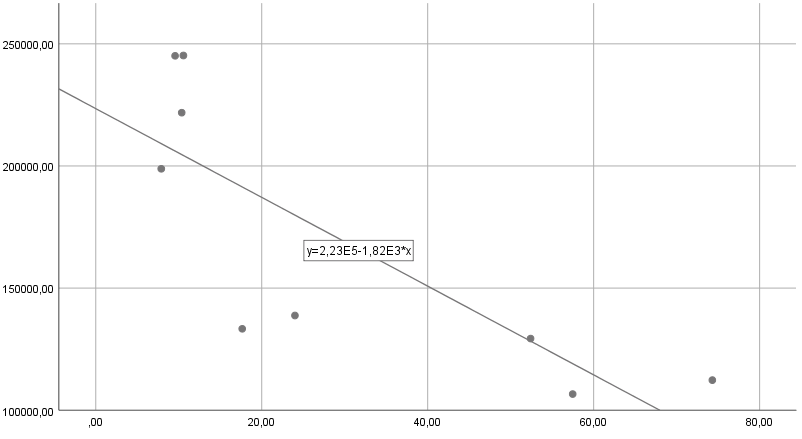


H. Male clozapine medicated

G. Male haloperidol medicated

I.-J. Hepatic aspartate aminotransferase (AST) and alanine aminotransferase (ALT) (abscissa) versus CYP1A1 raw volume (ordinate) of female haloperidol medicated SD rats


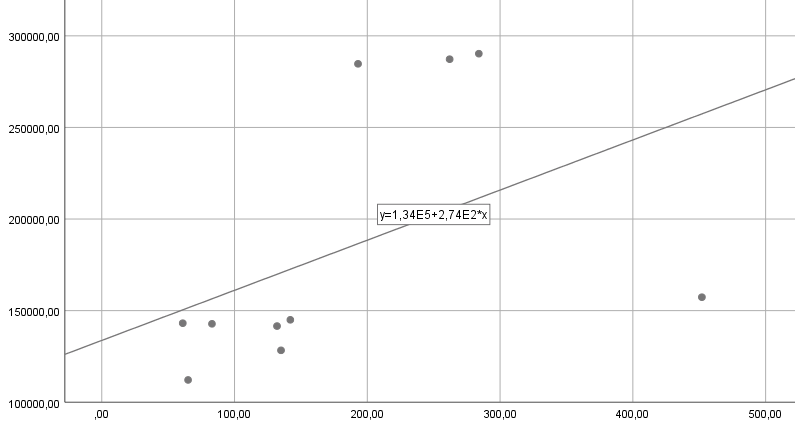

I. Female haloperidol medicated

J. Female haloperidol medicated


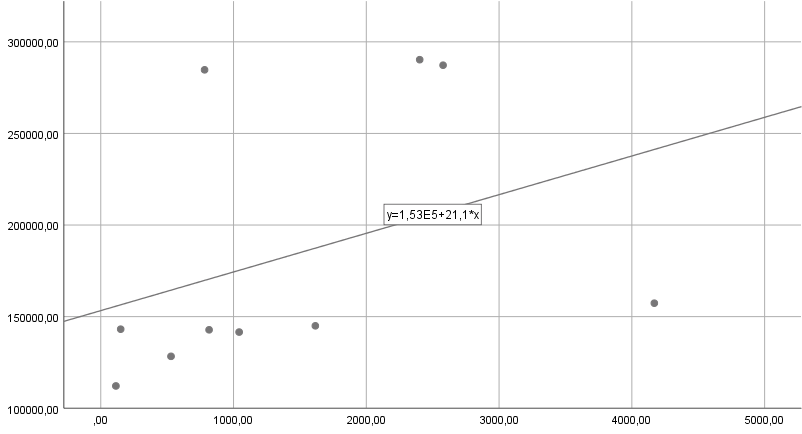

Supplement: Supplementary file 2 — Additional file 2. Supplementary figure 2. [file 40360_2021_544_MOESM2_ESM.docx]
